# Impact of Neodymium-Doped Yttrium Aluminum Garnet (ND-YAG) Posterior Capsulotomy Laser Treatment on Central Macular Thickness: A Prospective, Observational Study From a Tertiary Care Center

**DOI:** 10.7759/cureus.16242

**Published:** 2021-07-07

**Authors:** Moneeb Tariq, Kashif Iqbal, Bilal Inayat, Munib-ur Rehman, Waqas Zaheer, Malik A Shahid, Moiz Ahmed, Kiran Abbas

**Affiliations:** 1 Orthopedics, Layton Rehmatullah Benevolent Trust (LRBT) Free Eye Hospital, Lahore, PAK; 2 Ophthalmology, Layton Rehmatullah Benevolent Trust (LRBT) Free Eye Hospital, Lahore, PAK; 3 Ophthalmology, Khwaja M Safdar Medical College, Sialkot, PAK; 4 Medicine, Jinnah Postgraduate Medical Centre, Karachi, PAK; 5 Medicine and Surgery, Sindh Medical College, Karachi, PAK

**Keywords:** central macular thickness, posterior capsulotomy, posterior capsule opacification, nd-yag laser, optical coherence tomography (oct)

## Abstract

Introduction

Opacification of the posterior capsule is labeled as a secondary cataract. The objective of the current study was to assess central macular thickness (CMT) changes following neodymium-doped yttrium aluminum garnet (ND-YAG) posterior capsulotomy and to find out the correlation between CMT with the age, energy, and total shots utilized during the procedure.

Methodology

In this single-centered prospective observational study, 137 patients with a mean age of 57 ± 12.61 years, who had cataract surgery previously and were candidates for ND-YAG posterior capsulotomy were recruited through consecutive sampling. The study was conducted at Layton Rahmatulla Benevolent Trust (LRBT) Free Eye Hospital, Township, Lahore, Pakistan, between April 2020 to April 2021. The CMT, total energy, and sum total of shots used were recorded. The thickness of the central macula was measured preoperatively and postoperatively after two weeks. The paired sample t-test was used to find out any significant changes in CMT pre and two weeks postoperatively. The comparison of changes in CMT to age, energy, and the total number of shots was made through Pearson correlation. Means of CMT were compared using an independent sample t-test, at two weeks postoperatively among two energy groups.

Results

No statistically significant differences were found between preoperative and two weeks postoperative values of the CMT (P-value= 0.209). No significant difference was found in CMT statistically among the two energy groups (p=0.11). The patient's age, sum total of laser shots, and aggregate of laser energy did not have any significant correlation with CMT changes. The time period between cataract surgery and ND-YAG surgery showed a moderately positive correlation with a p-value of 0.01.

Conclusion

The current study revealed that ND-YAG capsulotomy does not affect the CMT significantly postoperatively. The patient's age, total energy applied, and the total number of laser shots used do not influence the macular thickness. However, the length of duration from the last cataract surgery to the current surgery was significantly associated with a change in the CMT.

## Introduction

Opacification of the posterior capsule is labeled as a secondary cataract. It is the biggest reported intricacy following modern cataract surgery [1]. Posterior capsule opacification (PCO) results from the proliferation of unsettled epithelial cells of the lens after surgery, as it is not suitable to remove all the epithelial cells during the surgical procedure [2]. The opacification observed in PCO varies in its intensity and can lead to multiple degrees of visual loss and complaints like glare disability, uniocular diplopia, compromised contrast sensitivity, and decline in visual acuity [3-4]. Neodymium-doped yttrium aluminum garnet (ND-YAG) laser surgery is the procedure of choice for cases of PCO with a reported success rate exceeding 95% [5]. ND-YAG posterior capsulotomy is a short procedure that removes an opacified capsule from a pseudoplastic eye. However, certain complications are also reported with this procedure like intraocular lens damage and dislocation (IOL), retinal detachment, and cystoid macular edema (CME) [6].

CME can develop following intraocular surgery due to inflammation of the retina and choroid. Different studies have reported the development of CME in 0%-4.3% of cases after ND-YAG posterior capsulotomy [7]. Vitreous cavity damage, along with the release of inflammatory mediators, which is elevated by damage to the blood-aqueous barrier, is considered to be the main cause of underlying macular edema and raised intraocular pressure following ND-YAG laser surgery [8]. According to previous studies, macular thickness is reduced when less energy (less than 80 mJ) is used in the procedure [9].

The current study is aimed to assess CMT changes preoperatively and at two weeks postoperatively after ND-YAG laser posterior capsulotomy when CME is expected to develop. In addition, we aimed to find out the CMT correlation with the quantum of energy used and the total sum of shots utilized during the procedure. Mixed evidence available on CMT changes after ND-YAG laser posterior capsulotomy established a need to explore the phenomenon in the local population.

## Materials and methods

A single-centered observational and prospective study was conducted at LRBT Free Eye Hospital, Township, Lahore, Pakistan. The study was performed by a set of expert surgeons. Consecutive sampling was used in this single-center study. The study was conducted after ethical approval from the hospital ethical committee. A total of 37 patients were enrolled in the study after informed consent. A total of 37 pseudophakic eyes with the complaint of significant PCO after cataract surgery were sampled for the current study. Patients managed with ND-YAG posterior capsulotomy were studied from July 2020 to December 2020.

Patients of all age groups and both genders who underwent non-complicated cataract surgery along with posterior chamber intraocular lens implant (IOL) and postoperative phacoemulsification were included in the study. A three-month postoperative duration was considered the minimum for inclusion in the study. On the other hand, patients with diagnosed glaucoma and anti-glaucoma drugs administration, uveitis, active eye inflammation, and trauma were excluded from the study.

After taking a complete history, each patient went through a detailed ocular examination that included slit-lamp biomicroscopic examination, fundus examination, best-corrected visual acuity measurement using the standard Snellen chart, and measurement of macular thickness with a spectral-domain-OCT (SD-OCT) device. After ophthalmologic examination, the patients underwent ND-YAG posterior capsulotomy procedure. One percent tropicamide was used for the dilation of pupils before surgery. The CMT was measured preoperatively and at two weeks of the post-procedure follow-up visit.

The quantum of total energy utilized and aggregate of shots used during the surgeries were recorded. These examinations and measurements of CMT were repeated after two weeks. Any possible complications were also taken into consideration.

The data were entered and analyzed using version 22 of the Statistical Package for the Social Sciences (SPSS; IBM Corp., Armonk, NY). All the descriptive and demographic data were presented as averages and standard deviation. The difference in CMT overtime (preop and two weeks postop) was calculated using the paired sample t-test. The difference in CMT after two weeks of procedure was also compared among groups based on energy used (less than 100 mJ and more than 100 mJ) and analyzed using an independent sample t-test. The possibility of any significant correlation among continuous variables was assessed using Pearson's correlation test. A P-value of less than 0.05 was considered statistically significant for all results.

## Results

The mean age of selected patients in the current study was 57 ± 12.61 years, ranging from 15 to 78. Out of 137 patients, 52 (37.96%) were male and 85 (62.04%) were females. The total duration from cataract surgery till ND-YAG laser posterior capsulotomy was no less than three months and up to 180 months maximum, with an average duration of 46.72 ± 39.78 months. The mean total shots count was 87 ± 52.41 with a range of a minimum of 14 and a maximum of 211 shots. The mean of the total energy used was 178.68 ± 148.80 mJ with a range of 7.70 to 530 mJ. 

The mean of CMT was 248.73±68.25 µm preoperatively. While the statistical mean of CMT at two weeks postoperative was 258.08±59.77 µm. With a p-value of 0.209, no significant difference was found statistically between preoperative and postoperative CMT (Table 1).

**Table 1:** Patient characteristics and central macular thickness changes at one week postoperatively SD=standard deviation, CMT=central macular thickness, Pa=paired sample t-test value as compared to preoperative measurement

Characteristics	Mean ± SD	Minimum-Maximum	P^a^
Age	57 ± 12.61	15 - 78	-
Duration between cataract surgery and laser (mo)	46.72 ± 39.78	3-180	-
Total laser energy (MJ)	178.68 ± 148.80	7.70 to 530	-
Total laser shots	87 ± 52.41	14-211	-
Preoperative central macular thickness (µm)	248.73 ± 68.25	23-440	-
2 weeks postoperative CMT (µm)	258.08 ± 59.77	120-443	0.209

Table 2 showed that no significant association was found between the patient's age, the aggregate of laser shots, the sum total of laser energy used, and the change in CMT preoperatively and two weeks postoperatively. Only time duration amid cataract surgery and ND-YAG laser surgery showed a significant correlation with a p-value of 0.01. A moderately positive correlation was observed (r = 0.46).

**Table 2:** Difference between preoperative versus postoperative central macular thickness P=2-tailed significance, Pearson correlation; ND-YAG=neodymium-doped yttrium aluminum garnet

Factor	Pearson correlation coefficient	P
Age	-0.25	0.13
Cataract surgery to ND-YAG laser surgery duration	0.46	0.01
Total laser energy	0.26	0.10
Total laser shots	0.27	0.09

The values of two weeks postoperative CMT were also compared among two energy level groups. One having energy levels less than 100 MJ and the second group having energy levels equal to or more than 100 mJ. No statistically significant difference was found in CMT between the two energy groups (p-value = 0.11). 

No patient developed a serious rise in intraocular pressure (IOP), CME, or severe anterior chamber reaction during the study.

## Discussion

ND-YAG laser capsulotomy is considered an accepted treatment for PCO that can develop as a result of cataract surgery. This non-invasive technique has replaced surgical capsulotomy [10]. Different studies have documented different results previously regarding CMT evaluation using OCT after ND-YAG laser posterior capsulotomy.

In the current study findings, no significant difference existed in CMT when compared preoperatively and two weeks postoperatively. Our results are in agreement with findings reported by a 2015 study conducted by Yuvacı et al., CMT was measured by Yuvacı et al. They measured CMT of 28 patients who were subjected to ND-YAG Posterior capsulotomy procedure for PCO. They measured changes in the thickness of the central macula following ND-YAG posterior capsulotomy at an interval of one day, then three days, then after two weeks, four weeks, and finally after 12 weeks, respectively. On the first postoperative day, an increase in CMT was reported. This increase was reverted on the third postoperative day and values on the second, fourth, and 12th weeks were similar to the values on the first day. They did not report any statistically significant change in CMT at any interval [11].

Another study conducted in 2017 by Yılmaz et al. in Turkey did not report any significant changes in CMT when measured in long-term follow-up. They measured CMT after one-week, four-week, three-month, six-month, and one-year intervals [12]. Insignificant changes in macular thickness were also reported by Casas et al. after three months of the procedure [13].

Contrary to the findings of our study, in 2014, Karahan et al. reported a significant increase in CMT one week after ND-YAG posterior capsulotomy, which reduced to pre-capsulotomy levels after one month [4]. Similarly, Abd-Elhafez in 2019 also reported a significant increase in macular thickness when compared preoperatively and one week postoperatively. Although, no difference was seen in macular thickness at three months postoperatively [14]. In the current study, no significant rise in CMT was observed two weeks postoperatively when compared to the preoperative values of CMT. The differences in findings of the current study, when compared with some previous studies, can be associated mainly with different follow-up periods of two weeks as compared to one-day, one-week, and one-month follow-up in the majority of previous studies. Moreover, different age groups and patient's characteristics, energy used, and time passed since last cataract surgery can also lead to differences in results.

In the current study, no significant difference in CMT was found between the pre versus postoperative groups. There was no significant difference observed between the amount of laser energy used. Contrary to the findings of the current study, in 2019, Parajuli et al. reported a significant increase in macular thickness after one hour and four weeks after ND-YAG capsulotomy, and its duration and severity were found related to the total amount of energy used [15].

In another study conducted in 2012, Ari et al. created two groups of patients based on energy levels. The first group was based on energy levels less than 80 mJ and the other group was based on energy more than 80 mJ. They outlined a rise in macular thickness in two groups selected at one-week and one-month follow-up with a higher thickness increase observed in the high energy group [9]. The differences with the current study can be attributed to different energy groups, different patient characteristics, and follow-up periods in both studies.

In 2010, Altiparmak et al. revealed that there was no significant modification in fovea thickness in relation to age, gender, energy, and shots used during the procedure [16]. These findings are in accordance with our study. Similarly, in 2011, Kara et al. also did not find any significant correlation between laser energy used and macular thickness postoperatively in their study [17].

Even though our study highlights the ND-YAG posterior capsulotomy laser as safe and efficacious cataract surgery, there were some limitations. The sample was collected from one center only and the sample size was small along with a short follow-up period, hence limiting the generalizability to a larger Pakistani population. Further multicenter studies on a larger scale should be conducted to ascertain these findings.

## Conclusions

The current study revealed that ND-YAG capsulotomy does not affect the central macular thickness significantly postoperatively. The patient's age, total energy applied, and the total number of laser shots used do not influence the macular thickness. However, the length of duration from the last cataract surgery to the current surgery was significantly associated with a change in the central macular thickness. Further multicenter studies on a larger scale should be conducted to ascertain these findings.
